# Unlocking employee creativity through digital leadership: a moderated mediation model of task innovation behavior and team collaboration climate

**DOI:** 10.3389/fpsyg.2026.1877187

**Published:** 2026-08-26

**Authors:** De’en Hou, Chen Lin, Ruofan Xu

**Affiliations:** 1 School of Economics and Management, Weifang University, Weifang, China; 2 School of Digital Commerce, Nanjing Vocational College of Information Technology, Nanjing, China; 3 School of Business, Shandong Jianzhu University, Jinan, China

**Keywords:** digital leadership, employee creativity, social cognitive theory, task innovation behavior, team collaboration climate

## Abstract

**Purpose:**

Prior research has linked digital leadership to employee creativity, but the task-level behavioral process underlying this relationship and the role of the surrounding team context have received less attention. Drawing on social cognitive theory, this study examines task innovation behavior as a mediator and tests whether team collaboration climate moderates its association with employee creativity.

**Design/methodology/approach:**

We collected data from 335 employee-supervisor dyads in China and tested the proposed model using hierarchical regression and bias-corrected bootstrapping.

**Findings:**

We found that digital leadership was positively associated with task innovation behavior, which in turn was positively related to employee creativity. The indirect association between digital leadership and employee creativity through task innovation behavior was significant. Team collaboration climate modestly strengthened the association between task innovation behavior and employee creativity, and the conditional indirect effect was stronger at higher levels of team collaboration climate.

**Practical implications:**

Organizations can support employee creativity by strengthening managers’ digital leadership capabilities. They should also encourage employees to experiment with work methods and develop task-specific solutions. A collaborative team climate can further help translate these task-level efforts into creative outcomes.

**Originality/value:**

The study extends current understanding of digital leadership and employee creativity by showing that task innovation behavior represents a proximal, task-level pathway. It further identifies team collaboration climate as a boundary condition for the relationship between task innovation behavior and employee creativity.

## Introduction

Employee creativity is an important source of organizational innovation and adaptability (Amabile, 1996; Bos-Nehles et al., 2017). By generating novel and useful ideas, employees contribute to improvements in products, services, and work processes and help organizations respond to competitive pressure and environmental uncertainty. Leadership is widely recognized as an important contextual influence on employee creativity because leaders shape employees’ access to resources, interpretation of the work environment, and willingness to experiment. Prior research has examined this influence through leader-member exchange and a range of established leadership approaches (Liao et al., 2010; Gong et al., 2009; Xu et al., 2022). Digital technologies have reshaped how work is organized and carried out. In this setting, supporting employee creativity requires leadership capabilities adapted to digitally enabled work.

Digital transformation has increased interest in digital leadership and its role in creativity and innovation (Avolio and Kahai, 2003; Shalley and Gilson, 2004; Nguyen et al., 2026). Digital leadership goes beyond using digital channels to perform traditional leadership activities. It combines traditional leadership functions with the ability to use digital technologies and data to guide change and coordinate work. These capabilities can help reshape work and support organizational transformation. They can also improve information sharing, resource coordination, and task execution while creating more flexible working conditions (Katz and Kahn, 1978; Baer and Oldham, 2006). Digital leadership may also shape leader–employee interactions and team coordination in collaborative and innovative work (Klus and Müller, 2021). By improving access to information and feedback, it can encourage employees to reconsider established ways of working. However, these benefits are not guaranteed. Digital practices may become constraining when employees experience them as monitoring or when digital demands exceed their skills, time, and available support (Deci and Ryan, 2000; Hobfoll, 1989; Ayyagari et al., 2011; Ertiö et al., 2024). Digital leadership may therefore be more conducive to creativity when employees have sufficient autonomy and resources to manage digital demands.

Existing research has explained the link between digital leadership and employee creativity through psychological processes or broader work behaviors. Studies have highlighted creative self-efficacy, psychological empowerment, and intrinsic motivation as important explanations of how leadership relates to creativity (Öngel et al., 2024; Gong et al., 2009; Zhang and Bartol, 2010). These perspectives help explain why employees may become more willing or confident to be creative, but they say less about what employees actually do during task execution. Social cognitive theory offers a useful basis for addressing this issue. It views behavior as the outcome of interactions among environmental cues, cognitive evaluations, and behavioral responses (Bandura, 2001). From this perspective, digital leadership can encourage employees to reconsider how they approach their tasks and explore alternative ways of working. Task innovation behavior therefore captures a more proximal behavioral pathway through which digital leadership is associated with employee creativity.

The study also considers the team context in which this behavioral pathway operates. Leadership effects can vary across team and organizational settings, making contextual conditions an important issue in leadership research (Oc, 2018; Fischer et al., 2017; Hughes et al., 2018). Team collaboration climate reflects shared perceptions of open communication, mutual support, knowledge sharing, and joint problem solving. A collaborative climate can give employees opportunities to exchange ideas, obtain feedback, and refine task-level innovations (Anderson and West, 1998; Hülsheger et al., 2009; West and Sacramento, 2006; Somech and Drach-Zahavy, 2013). These conditions may help employees develop task innovation behavior into more creative outcomes. Team collaboration climate is therefore relevant to the link between task innovation behavior and employee creativity. Yet this team-level condition has received limited attention in research on digital leadership.

**Figure 1:**
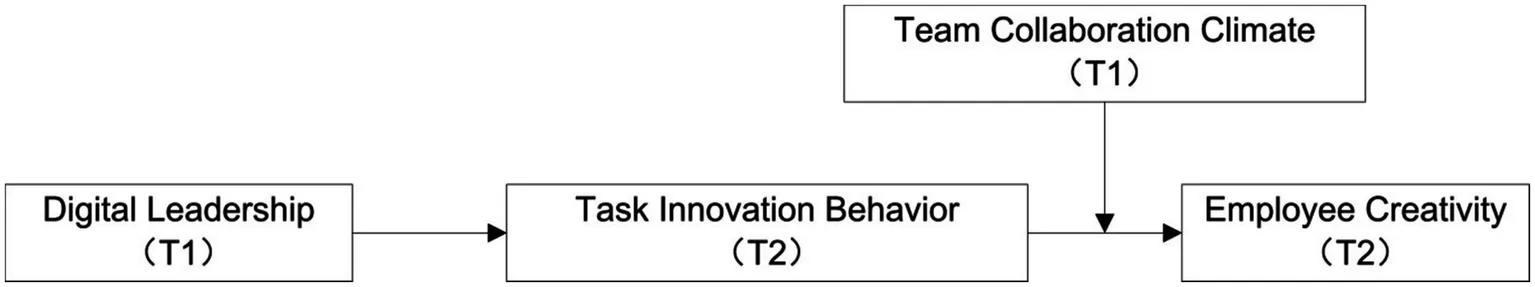
Theoretical model.

This study examines task innovation behavior as a mediator between digital leadership and employee creativity. It also tests whether team collaboration climate moderates the link between task innovation behavior and employee creativity. The model focuses on how employees respond to digital leadership through task-level innovation efforts and how the team environment influences the extent to which these efforts are associated with creativity. This approach complements prior research centered on psychological mechanisms and broader innovation outcomes (Hoever et al., 2012; Somech and Drach-Zahavy, 2013). Figure 1 presents the proposed model.

## Theoretical background and hypotheses

### Digital leadership and task innovation behavior

Digital leadership overlaps with established leadership approaches but remains conceptually distinct. It shares transformational leadership’s emphasis on change and empowering leadership’s support for autonomy. It also resembles innovation leadership in its emphasis on new ideas (Gong et al., 2009; Zhang and Bartol, 2010; Hughes et al., 2018). Its distinctive features lie in digital vision, technological competence, and the use of data and digital resources to coordinate work and guide technology-enabled change (Cortellazzo et al., 2019; Klus and Müller, 2021). In this study, digital leadership refers to leaders’ capacity to direct and support such change through the effective use of digital technologies.

Digital leadership also differs from related technology-based leadership constructs in its primary focus. E-leadership broadly concerns the interplay between leadership and digital technologies, whereas virtual leadership focuses more specifically on leading employees or teams across geographical distance through technology-mediated communication (Avolio et al., 2000; Bauwens and Cortellazzo, 2025). By contrast, digital leadership centers on the use of digital capabilities and resources to reshape work and guide organizational transformation.

Digital transformation expands leadership beyond traditional planning and control. Leaders must also use digital technologies to reshape how work is organized and carried out. Early work on e-leadership showed that information technology can change how and where leadership is enacted, making leadership increasingly technology-mediated (Avolio et al., 2000; Avolio and Kahai, 2003). Later studies placed greater emphasis on digital vision, resource integration, and technology-enabled coordination. These capabilities can support employees’ adaptability, initiative, and willingness to innovate (Cortellazzo et al., 2019; Gierlich-Joas et al., 2020; Zhu et al., 2022).

Task innovation behavior is conceptualized here as a task-specific form of innovative work behavior. Whereas innovative work behavior spans opportunity exploration, idea generation, idea championing, and implementation across the broader work role (De Jong and Den Hartog, 2010; Farrukh et al., 2023), task innovation behavior centers on employees’ efforts to improve work methods, revise procedures, and develop solutions while carrying out their tasks. It is also conceptually distinct from job crafting, which concerns changes to the boundaries of one’s work, and from proactive behavior, which covers a broader range of self-initiated and future-oriented actions (Wrzesniewski and Dutton, 2001; Parker et al., 2010). This task-level focus is particularly relevant because digital leadership can reshape everyday work through the use of digital technologies. Task innovation behavior thus represents a proximal behavioral response through which employees can act on the opportunities and cues provided by digital leadership.

Social cognitive theory suggests that individual behavior develops through the interaction between environmental cues and cognitive processes (Bandura, 1986). In the workplace, leaders act as salient social referents whose actions signal which behaviors are supported. Digital leaders can encourage innovation by demonstrating how digital technologies can be used to solve work problems and by showing openness to experimentation. They can also make task experimentation easier by improving employees’ access to information and relevant resources. These conditions can strengthen employees’ perceived self-efficacy and control in task-related innovation (Bandura, 1977; Spreitzer, 1995). When employees observe that their leaders use digital technologies to solve complex problems and encourage innovation, they are more likely to believe that their own tasks can be improved through new methods. This belief can lead them to engage in process optimization, method innovation, and problem reframing. The beneficial effects of digital leadership may weaken when employees experience digital practices as intrusive monitoring, lack the competence to use digital technologies effectively, or face digital demands that exceed their available time and support. Under these conditions, lower perceived autonomy and greater resource strain may reduce employees’ willingness or capacity to experiment with their tasks (Deci and Ryan, 2000; Hobfoll, 1989). Although these conditions are theoretically relevant, they are not examined directly in the present study, which focuses on the supportive and resource-enabling aspects of digital leadership. Accordingly, the following hypothesis is proposed.

*H1*: Digital leadership is positively related to task innovation behavior.

### Task innovation behavior and employee creativity

Employee creativity concerns the generation of novel and useful ideas, whereas innovative behavior extends beyond idea generation to their promotion and implementation (Amabile, 1996). Creativity and innovation are closely connected but place emphasis on different stages of this process. Creativity centers more on idea generation, while innovation gives greater attention to idea promotion and implementation (Anderson et al., 2014). The dynamic componential model views creativity as an evolving process shaped by employees’ progress and responses to feedback (Amabile and Pratt, 2016). Employees who repeatedly explore new methods and solutions in their tasks may therefore be more likely to generate and refine creative ideas.

Task innovation behavior may support employee creativity by prompting employees to explore new approaches and integrate knowledge during task execution. Employees engaged in task innovation often search for better ways to address work problems by drawing on available information and considering alternative solutions. Such activities can strengthen the cognitive processes involved in developing creative ideas (Shalley and Gilson, 2004). Task innovation also goes beyond generating ideas. Employees put new approaches into practice and refine them in response to feedback. Related research on job crafting suggests that proactively adjusting aspects of one’s work can create better conditions for creativity (Wrzesniewski and Dutton, 2001). Active involvement in the creative process is also associated with stronger creative performance (Zhang and Bartol, 2010). Sustained engagement in creative tasks can also strengthen creative self-efficacy and support further idea development (Tierney and Farmer, 2011). These arguments suggest that task innovation behavior provides a behavioral pathway linking employees’ task-level efforts with creative outcomes. Accordingly, the following hypothesis is proposed:

*H2*: Task innovation behavior is positively related to employee creativity.

### The mediating role of task innovation behavior

Prior research generally supports the positive role of digital leadership in employee creativity, but the relationship is unlikely to operate as a simple direct effect. It is more likely to work through behavioral processes embedded in daily work. Studies on digital leadership and innovation-related outcomes have examined mechanisms such as job crafting, organizational learning, innovation capability, and digital literacy (Zhu et al., 2022; Ahmed et al., 2024; Wang et al., 2025). These studies suggest that digital leadership often needs to be translated into employees’ proactive responses before it appears in creativity-related outcomes. Digital leadership therefore does more than create a general innovation-oriented climate. It can also encourage employees to take substantive innovation actions in specific tasks.

From the perspective of social cognitive theory, task innovation behavior can connect digital leadership with employee creativity because it captures the behavioral process by which contextual cues become creative outcomes. Digital leaders signal support for change and innovation by using digital technologies in decision making and encouraging experimentation. Employees may respond to these signals by trying new methods, revising existing routines, and testing alternative solutions in their work tasks. Once task-level innovation is activated, employees gain more experience in problem reframing, solution experimentation, and feedback-based adjustment. These experiences can support the development of creativity (Parker et al., 2010).

Digital leaders can foster task-level innovation by using digital technologies to improve coordination and resource access. These conditions give employees greater scope to experiment with how they perform their tasks (Wrzesniewski and Dutton, 2001; Tims et al., 2012; Petrou et al., 2012). These adjustments may then develop into higher levels of creativity. Existing research has shown that digital leadership can promote employee creativity through job crafting and can improve innovation performance through task and cognitive crafting (Zhu et al., 2022; Wang et al., 2025). This study therefore expects digital leadership to foster employee creativity by stimulating employees’ innovation behavior during task execution. Accordingly, the following hypothesis is proposed:

*H3*: Task innovation behavior mediates the relationship between digital leadership and employee creativity.

### The moderating role of team collaboration climate

Task innovation behavior reflects employees’ efforts to find better ways of performing their work tasks. However, such behavior does not always become employee creativity. Social cognitive theory suggests that outcomes are shaped by the dynamic interaction among personal cognition, behavior, and environmental factors (Bandura, 2001). Whether employees can develop their task-level innovative attempts into creative outcomes depends not only on their own behavior but also on whether the team environment provides support and reinforcement. Creativity research has also shown that employee creativity is embedded in the work environment and social interaction. A supportive team environment can foster employee creativity by enabling employees to exchange and refine new ideas (Amabile, 1996; Anderson and West, 1998; Liu et al., 2020).

A strong team collaboration climate creates a supportive setting for employees to exchange ideas and coordinate their work. In such teams, ideas emerging from task execution are more likely to be discussed, refined, and supported by colleagues. These ideas can then be refined and developed into creative outcomes. In contrast, when the collaboration climate is weak, task innovation behavior may remain largely individual because employees have fewer opportunities to exchange ideas and refine their approaches with colleagues. Research on team innovation climate also shows that supportive and participative team conditions help employees develop innovative behavior into concrete outcomes (Foss et al., 2013; Wang, 2017; You et al., 2022). It is therefore reasonable to expect that the positive relationship between task innovation behavior and employee creativity will be stronger when team collaboration climate is high. Accordingly, the following hypothesis is proposed:

*H4*: Team collaboration climate positively moderates the relationship between task innovation behavior and employee creativity, such that the positive effect of task innovation behavior on employee creativity is stronger when team collaboration climate is high.

### The moderated mediation role of team collaboration climate

The preceding arguments imply that team collaboration climate may also condition the indirect effect of digital leadership on employee creativity through task innovation behavior. Digital leadership first needs to be translated into employees’ innovative actions in their work tasks. Whether these actions can become creative outcomes depends on the collaborative support, knowledge sharing, and interactional safety available in the team. Amabile and Pratt (2016) emphasize that creativity and innovation develop through employees’ interactions with their work environment and ongoing task experience. Team collaboration climate is therefore not only a context in which task innovation behavior becomes effective. It may also shape the overall strength of the pathway from digital leadership to employee creativity.

When team collaboration climate is high, colleagues are more likely to provide input that helps employees refine and develop new ideas. Task innovation behavior can then be sustained and transformed into stronger creativity-related outcomes (Zhang and Bartol, 2010). When team collaboration climate is low, digital leadership may still encourage task innovation behavior, but limited support from colleagues can make it harder for employees to develop these efforts into creative outcomes. Related studies suggest that digital leadership is linked to innovation outcomes through employees’ adaptive responses to work. The effectiveness of these pathways may depend on the support available in the surrounding work context (Zhu et al., 2022; Ahmed et al., 2024; Wang et al., 2025). Thus, the indirect effect of digital leadership on employee creativity through task innovation behavior should be stronger when team collaboration climate is high and weaker when it is low. Accordingly, the following hypothesis is proposed:

*H5*: Team collaboration climate moderates the indirect effect of digital leadership on employee creativity through task innovation behavior, such that this indirect effect is stronger when team collaboration climate is high.

## Methods

### Participants and procedures

To reduce concerns about common method bias, we combined temporal separation with source separation (Podsakoff et al., 2003). We recruited participants through MBA classes and alumni networks at a university in Jinan, China, using convenience sampling. All focal respondents were full-time employees who were enrolled in the university’s part-time MBA program or had completed it previously. We included only respondents who had an identifiable immediate supervisor willing to complete the matched supervisor questionnaire. Participants therefore took part in the study as employees rather than as university students. The sample spanned several industries, including services, biotechnology, manufacturing, food processing, and agriculture.

Before the survey, participants were informed that the research was for academic purposes only. They were also told that the questionnaires would be anonymous and that the data would be used only for statistical analysis, not for organizational evaluation or performance appraisal. Employees and their immediate supervisors completed the questionnaires independently to minimize response contamination between matched respondents. In the first stage, employees evaluated their immediate supervisors’ digital leadership and the team collaboration climate of their work units. They also provided an identification code for later matching. In the second stage, immediate supervisors evaluated employees’ task innovation behavior and creativity. The research team matched the two waves of responses using the identification codes and formed employee-supervisor paired data. After screening for patterned responses, missing key information, inconsistent answers, and unmatched records, the final sample consisted of 335 valid dyads. The final sample consisted of 335 employees drawn from the service sector (20.9%), manufacturing (20.6%), biotechnology (17.9%), food processing (13.7%), agriculture (10.7%), and other industries (16.1%). Of the participants, 32.5% were male and 67.5% were female. Most participants were aged 40 years or younger, and 71.0% held a bachelor’s degree. Regarding organizational tenure, 92.9% had worked in their current organization for 5 years or less, while 7.2% had 6 to 10 years of tenure.

To assess the adequacy of the final sample, a sensitivity analysis was conducted using G*Power 3.1. For a fixed-model multiple regression test of the increase in R^2^, with one tested predictor, eight predictors in the full model, *α* = 0.05, power of 0.80, and 𝑁=335, the minimum detectable effect size was 𝑓^2^ = 0.024. This result indicates that the sample was sufficiently sensitive to detect a small incremental effect. The moderated mediation effect was further assessed using bias-corrected bootstrap confidence intervals.

### Measures

All focal constructs were measured with established scales from prior research. Minor wording changes were made to fit the Chinese organizational context. Except for the control variables, all items used a five-point Likert scale ranging from 1 = strongly disagree to 5 = strongly agree. Before the formal survey, the English scales were translated and back-translated to improve conceptual equivalence between the Chinese and original versions.

*Digital leadership*: Digital leadership was assessed with the six-item scale developed by Zeike et al. (2019). The scale captures leaders’ digital skills and their ability to manage digital transformation. It fits this study because it reflects leaders’ use of digital technologies, support for digital change, and guidance of teams in a digital environment. The items were slightly adapted to capture employees’ perceptions of their immediate supervisors. Sample items were: My immediate supervisor can effectively use digital tools for communication and management; and My immediate supervisor can help the team adapt to digital change.

*Task innovation behavior*: We assessed task innovation behavior using the 10-item innovative work behavior scale developed by De Jong and Den Hartog (2010). We adapted the item wording to focus specifically on employees’ current work tasks. In this study, the measure serves as a task-focused operationalization of innovative work behavior rather than as a distinct construct. The adapted items capture opportunity exploration, idea generation, idea championing, and implementation during task execution. Example items include “This employee actively looks for ways to improve their current work tasks,” “This employee tries to apply new ideas to their work tasks,” and “This employee promotes the implementation of new solutions in their work.”

*Team collaboration climate*: Team collaboration climate was measured with items drawn from the short version of the Team Climate Inventory developed by Anderson and West (1998). The Team Climate Inventory is widely used in team climate research and includes shared vision, participative safety, task orientation, and support for innovation. Given the focus of this study, the selected items captured open communication, mutual support, collaborative problem solving, and cooperation in innovation-related activities. Sample items were: Team members are willing to share work-related information; Team members collaborate fully when facing problems; and The team is supportive of new ideas.

*Employee creativity*: Employee creativity was measured with the scale developed by Zhou and George (2001). This scale is widely used in organizational behavior research and captures employees’ ability to generate novel and useful ideas, improve work methods, and develop feasible solutions. Prior studies have often used the scale to assess whether employees propose new and practical ideas for improving performance and achieving work goals. In this study, immediate supervisors evaluated employee creativity, which helped reduce concerns about common source bias.

*Control* var*iables*: Following prior research on employee innovation and creativity, we controlled for gender, age, education, and organizational tenure (Amabile et al., 1996; Shalley and Gilson, 2004; Jaiswal and Dhar, 2015). Gender was coded as 1 = male and 2 = female. Age was coded into five ordered categories: 1 = 25 years or younger, 2 = 26–30 years, 3 = 31–35 years, 4 = 36–40 years, and 5 = 41 years or older. Education ranged from 1 = high school or below to 5 = doctoral degree, while organizational tenure ranged from 1 = less than 1 year to 3 = 6–10 years. These controls account for differences in demographic background, knowledge, work experience, and familiarity with organizational routines.

## Results

### Reliability and validity tests

The reliability of the measures was examined before hypothesis testing. Cronbach’s alpha values were 0.751 for digital leadership, 0.675 for task innovation behavior, 0.868 for employee creativity, and 0.736 for team collaboration climate. Digital leadership, employee creativity, and team collaboration climate showed acceptable internal consistency, whereas the value for task innovation behavior was comparatively modest. We therefore conducted additional reliability checks for this measure. Its composite reliability was 0.668, McDonald’s omega was 0.686, and the mean inter-item correlation was 0.170. Although the first two coefficients were modest, the mean inter-item correlation falls within the range considered appropriate for a broad measure containing related but non-redundant behavioral components (Cortina, 1993; Clark and Watson, 1995). We retained all 10 items to preserve coverage of opportunity exploration, idea generation, idea championing, and implementation. Results involving task innovation behavior should therefore be interpreted with appropriate caution.

The correlations among the four core variables were in the expected directions. This pattern indicates that the constructs were meaningfully related and provides an initial basis for the following hypothesis tests. Overall, the data met the basic requirements for further empirical analysis.

Confirmatory factor analysis was used to test whether the four core constructs were empirically distinct. As Table 1 shows, the four-factor model fit the data best. Its chi-square/df value was 1.921, below the commonly used threshold of 3. The GFI and AGFI values were 0.921 and 0.906, respectively, and the CFI was 0.967. The RMSEA was 0.015, indicating very good fit. The fit indices became weaker for the three-factor and two-factor models, and the one-factor model showed the poorest fit. These results support the discriminant validity of the constructs (Fornell and Larcker, 1981).

**Table 1:** Confirmatory factor analysis model fit indices.

Model	Factor	Chi-square/df	GFI	AGFI	CFI	RMSEA	TLI
Four-factor model	DL, TIB, EC, TCC	1.921	0.921	0.906	0.967	0.015	0.958
Three-factor model	DL + TIB, EC, TCC	2.315	0.896	0.873	0.903	0.043	0.938
Two-factor model	DL + TIB, EC + TCC	3.015	0.812	0.789	0.897	0.076	0.899
One-factor model	DL + TIB + EC + TCC	4.568	0.765	0.732	0.789	0.091	0.807

Chi-square/df, chi-square divided by degrees of freedom; GFI, goodness-of-fit index; AGFI, adjusted goodness-of-fit index; CFI, comparative fit index; RMSEA, root mean square error of approximation; TLI, Tucker-Lewis index.

### Common method bias test

Harman’s single-factor test was used to assess common method bias. The first unrotated factor explained 23.10% of the total variance, which is below the commonly used 40% threshold (Podsakoff et al., 2003). This result suggests that common method bias was not likely to be a serious threat to the findings.

### Descriptive statistics and correlation analysis

Table 2 reports the descriptive statistics and correlations. The mean values of digital leadership, task innovation behavior, employee creativity, and team collaboration climate were 3.585, 3.785, 3.734, and 3.758, respectively. All four mean values were above the midpoint of the scale.

**Table 2:** Descriptive statistics and correlations.

Variables	Mean	SD	Cα	Gender	Age	Education	Org. tenure	DL	TIB	EC	TCC
Gender	1.675	0.469		—							
Age	1.200	0.717		−0.189***	—						
Education	2.916	0.750		0.093	0.689***	—					
Org. tenure	1.603	0.620		−0.219***	0.341***	0.244***	—				
DL	3.585	0.558	0.751	−0.201***	−0.188***	−0.113*	−0.187***	**0.612**			
TIB	3.785	0.414	0.675	0.092	−0.261***	−0.170**	−0.154**	0.584***	**0.626**		
EC	3.734	0.737	0.868	0.151**	0.013	0.276***	0.245***	0.224***	0.444***	**0.591**	
TCC	3.758	0.396	0.736	−0.136*	−0.029	−0.071	0.149**	0.316***	0.535***	0.503***	**0.563**

*N* = 335. Bold diagonal values are the square roots of AVE. Values below the diagonal are Pearson correlation coefficients. *p < 0.05, **p < 0.01, and ***p < 0.001.

The correlations were consistent with the proposed relationships. Digital leadership was positively related to task innovation behavior (*r* = 0.584, *p* < 0.001), employee creativity (*r* = 0.224, *p* < 0.001), and team collaboration climate (*r* = 0.316, *p* < 0.001). Task innovation behavior was positively related to employee creativity (*r* = 0.444, *p* < 0.001) and team collaboration climate (*r* = 0.535, *p* < 0.001). Team collaboration climate was also positively related to employee creativity (*r* = 0.503, *p* < 0.001). These correlations provide an initial view of the bivariate associations among the focal variables.

### Hypothesis testing

Hierarchical regression analysis was used to test the hypotheses (Baron and Kenny, 1986). Table 3 reports unstandardized coefficients from seven models. Models 1 and 2 used task innovation behavior as the dependent variable, whereas Models 3–7 used employee creativity as the dependent variable. Gender, age, education, and organizational tenure were entered as controls in all models.

**Table 3:** Hierarchical regression results for direct and indirect effects.

Variables	TIB	TIB	EC	EC	EC	EC	EC
Model	M1	M2	M3	M4	M5	M6	M7
Control variables							
Gender	0.029	0.201***	0.154	0.314***	0.142	0.248***	0.296***
Age	−0.135**	−0.029	−0.394***	−0.296***	−0.271***	−0.307***	−0.282***
Education	0.003	−0.055	0.454***	0.401***	0.448***	0.476***	0.456***
Org. tenure	−0.045	0.035	0.338***	0.413***	0.383***	0.260***	0.270***
Independent variable							
DL		0.459***		0.424***	0.032	0.041	0.048
Mediating variable							
TIB					0.855***	0.309***	0.309***
Moderating variable							
TCC						0.960***	0.999***
Interaction							
TIB × TCC							0.278*
R^2^	0.074	0.404	0.207	0.296	0.433	0.601	0.606
Adjusted *R*^2^	0.063	0.395	0.197	0.285	0.422	0.593	0.597
*F*-value	6.572***	44.642***	21.516***	27.629***	41.704***	70.506***	62.774***

*N* = 335; *p < 0.05, **p < 0.01, and ***p < 0.001.

Task innovation behavior was first entered as the dependent variable. After gender, age, educational level, and organizational tenure were controlled, digital leadership was positively associated with task innovation behavior (*B* = 0.459, *p* < 0.001). This result indicates that employees are more likely to engage in innovative actions during task execution when they perceive higher levels of digital leadership. H1 was therefore supported. The result is shown in Model 2 of Table 3.

Employee creativity was then entered as the dependent variable. After the control variables and digital leadership were included, task innovation behavior was added to the model. Task innovation behavior was positively associated with employee creativity (B = 0.855, *p* < 0.001). H2 was therefore supported. The result is reported in Model 5 of Table 3.

The coefficients of the control variables varied across the models. Models 1 and 2 predicted task innovation behavior, whereas Models 3–7 predicted employee creativity; coefficients across these two sets of models are therefore not directly comparable. Within the employee creativity models, age was negatively associated with creativity, whereas education and organizational tenure showed positive associations. The coefficient for gender varied with model specification. Because these variables were included as controls rather than focal predictors, their effects are treated as ancillary findings.

The mediating effect of task innovation behavior was tested with a bootstrapping procedure using 5,000 resamples and 95% confidence intervals (Preacher and Hayes, 2004). As Table 4 shows, the indirect effect of digital leadership on employee creativity through task innovation behavior was 0.392, and the 95% confidence interval was [0.296, 0.493]. Because this interval did not include zero, the indirect effect was significant.

**Table 4:** Bootstrap results for mediation effects.

Effect type	Effect size	Boot SE	95% CI lower bound	95% CI upper bound
Total effect	0.424	0.069	0.293	0.566
Direct effect	0.032	0.073	−0.100	0.182
Indirect effect	0.392	0.050	0.296	0.493

Bootstrap size = 5,000.

The direct effect of digital leadership on employee creativity was 0.032, with a 95% confidence interval of [−0.100, 0.182]. Although the direct effect was not statistically significant, this result should not be interpreted as evidence that the direct effect was exactly zero. The findings therefore identify task innovation behavior as an important mediating pathway, while a small residual direct effect cannot be ruled out. H3 was supported.

The moderating effect of team collaboration climate was tested by mean-centering task innovation behavior and team collaboration climate and entering their interaction term into the regression model (Baron and Kenny, 1986). After the relevant variables were controlled, the interaction between task innovation behavior and team collaboration climate was positive and statistically significant (B = 0.278, *p* = 0.045). Model 7 of Table 3 reports this result. The finding indicates that team collaboration climate strengthens the positive relationship between task innovation behavior and employee creativity. H4 was supported.

**Figure 2:**
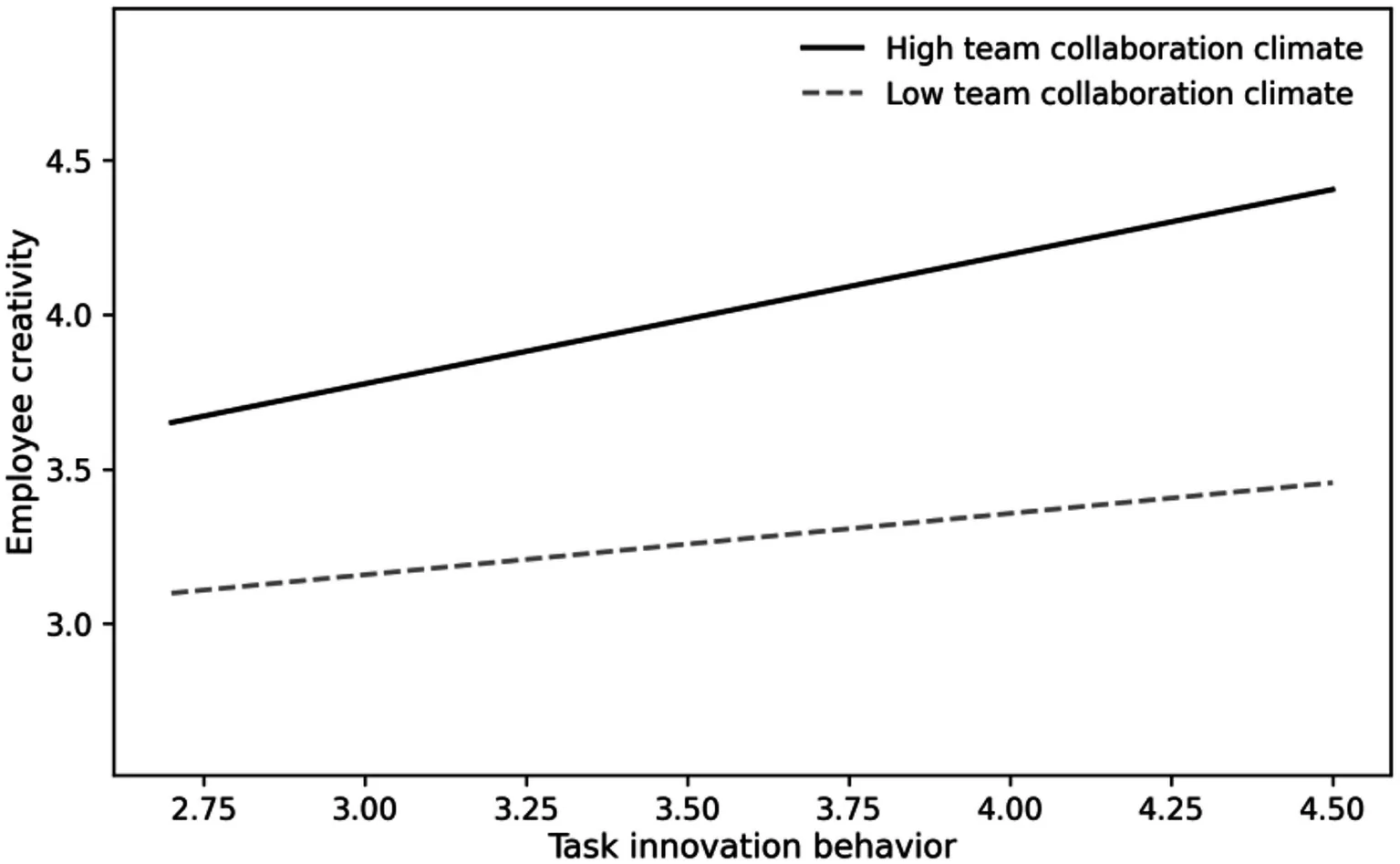
Moderating effect of team collaboration climate on the relationship between task innovation behavior and employee creativity.

To probe the interaction, we estimated simple slopes at high and low levels of team collaboration climate. As shown in Table 5, task innovation behavior was positively associated with employee creativity when team collaboration climate was high (B = 0.419, *p* < 0.001). The corresponding association was weaker and did not reach conventional statistical significance when team collaboration climate was low (B = 0.198, *p* = 0.067). Although the moderation was modest, the pattern suggests that a collaborative team environment helps employees develop task-level innovation efforts into creative contributions rather than serving as a decisive condition. Its practical value lies in creating opportunities for employees to discuss emerging ideas, receive feedback, and improve proposed solutions. Figure 2 presents the interaction plot.

**Table 5:** Simple slope results for the moderating effect of team collaboration climate.

Moderator	Level	Simple slope	Standard error	*p*
Team Collaboration Climate	HIGH (M + 1 SD)	0.419	0.115	<0.001
	LOW (M − 1 SD)	0.198	0.117	0.067

Bootstrap size = 5,000. High and low team collaboration climates refer to one standard deviation above and below the mean, respectively.

The moderated mediation effect was then examined. Table 6 presents the bootstrap results based on 5,000 resamples. When team collaboration climate was high, the indirect effect of digital leadership on employee creativity through task innovation behavior was 0.192, with a 95% confidence interval of [0.101, 0.280]. When team collaboration climate was low, the indirect effect was 0.091, with a 95% confidence interval of [0.006, 0.174]. Both indirect effects were positive and significant, but the effect was stronger under a high level of team collaboration climate.

**Table 6:** Bootstrap results for moderated mediation.

Moderator	Level	Indirect effect	Standard error	95% CI
Team Collaboration Climate	HIGH (M+1 SD)	0.192	0.046	(0.101, 0.280)
	LOW (M−1 SD)	0.091	0.043	(0.006, 0.174)
	Index of moderated mediation	0.128	0.049	(0.035, 0.225)

Bootstrap size = 5,000.

The index of moderated mediation was 0.128, and its 95% confidence interval was [0.035, 0.225]. Because the interval did not include zero, the moderated mediation effect was significant (Hayes, 2015). This finding indicates that team collaboration climate not only conditions the effect of task innovation behavior on employee creativity, but also strengthens the mechanism through which digital leadership promotes employee creativity via task innovation behavior. H5 was supported.

## Discussion

This study examined how digital leadership relates to employee creativity through task innovation behavior and whether team collaboration climate shapes this process. Digital leadership was positively associated with task innovation behavior, which in turn was linked to higher supervisor-rated employee creativity. The significant indirect effect is consistent with task innovation behavior serving as a task-level behavioral pathway. Team collaboration climate modestly strengthened the link between task innovation behavior and employee creativity. The findings suggest that digital leadership is more likely to be associated with creativity when employees use digital resources and leadership cues to improve how they perform their tasks. A collaborative team environment may further support this process by giving employees opportunities to discuss, evaluate, and refine emerging ideas. The results indicate that this behavioral pathway varies across team contexts rather than operating with the same strength in all settings.

### Theoretical implications

First, this study provides a more specific account of the link between digital leadership and employee creativity (Zhu et al., 2022; Öngel et al., 2024; Wang et al., 2025). Prior studies have documented positive associations between digital leadership and creativity or other innovation-related outcomes. This evidence establishes the relevance of digital leadership, but provides a relatively broad picture of its connection with employee creativity. The present study builds on this work by examining the relationship as more than a simple direct association. It therefore shifts attention from whether digital leadership is related to creativity toward a more precise explanation of how this link operates in digitally enabled work.

Second, this study offers a more behavior-focused explanation of the link between digital leadership and employee creativity. Prior research has mainly examined psychological mechanisms, including psychological empowerment, intrinsic motivation, creative self-efficacy, and learning orientation (Tierney and Farmer, 2002; Gong et al., 2009). These mechanisms help explain why employees become more willing or confident to be creative, but provide less insight into what they actually do during everyday work. Task innovation behavior captures concrete efforts to improve work methods, revise procedures, and develop new solutions. This focus is consistent with research showing that creativity develops as employees address work problems and refine emerging ideas in response to experience and feedback (Anderson et al., 2014; Amabile and Pratt, 2016). Our findings point to task innovation behavior as one proximal route linking digital leadership with employee creativity. The significant indirect effect supports this interpretation, but the nonsignificant residual direct effect does not imply complete mediation. Other psychological and social pathways may also be involved. The behavioral explanation offered here therefore complements rather than replaces existing accounts.

Third, this study contributes to digital leadership research by identifying team collaboration climate as an important boundary condition. Innovation research has repeatedly shown that creativity is embedded in the team environment rather than produced in isolation. Team climate, collaborative support, and knowledge sharing all shape the generation, refinement, and implementation of new ideas (Edmondson, 1999; Hülsheger et al., 2009). However, digital leadership studies have tended to emphasize leaders’ digital skills and digital vision, while giving less attention to the social context in which leadership effects unfold. The present results show that team collaboration climate strengthens both the relationship between task innovation behavior and employee creativity and the indirect effect of digital leadership on employee creativity through task innovation behavior. These findings indicate that the task-level pathway becomes stronger when teams provide open communication, mutual support, and collaborative engagement. This study brings digital leadership and team climate research together by showing that the association between leadership and employee creativity varies with the surrounding team context.

### Potential adverse effects of digital leadership

The positive associations found in this study should not be taken to mean that digital leadership always benefits employee creativity. Its effects may depend on how employees experience digital practices and whether they have sufficient resources to meet digital demands. Practices that increase autonomy and strengthen employees’ digital competence can make experimentation easier and support the development of new ideas. However, the same practices may become constraining when employees perceive them as monitoring, face expectations of constant availability, or lack the time and skills needed to adapt. From the perspectives of self-determination theory and conservation of resources theory, these conditions may reduce autonomy and increase technostress or cognitive load. Digital leadership may therefore be more supportive of creativity when employees have adequate autonomy, competence, time, and organizational support. Future research could examine when digital leadership shifts from an enabling influence to a source of constraint.

### Practical implications

The findings also offer practical implications. Organizations that seek to improve employee creativity should not rely only on general calls for innovation. They should pay closer attention to employees’ innovation-related behavior in specific work tasks. The results show that task innovation behavior is an important pathway linking digital leadership with employee creativity. Creativity in a digital management context does not arise automatically. It develops when employees continue to improve methods, optimize processes, and rethink work problems. Organizations can support this process through task empowerment, job redesign, process feedback, and appropriate innovation incentives. These practices give employees more autonomy and a reasonable space for experimentation, which helps them translate innovation awareness into daily work actions.

Organizations should also strengthen digital leadership capabilities among managers. The value of digital leadership is not limited to technology adoption or the use of digital tools. It also depends on whether leaders can communicate a digital vision, provide resources, encourage open communication, and support employees in exploring new ways of working. In this sense, digital leadership is more likely to support creativity when it helps employees translate digital change into task-level innovation efforts. Organizations should include digital leadership development in managerial training and focus on digital cognition, technology-enabled communication, resource integration, and innovation guidance.

The findings are also relevant for digital transformation practice. Many organizations invest heavily in digital systems, platform construction, and process redesign, but give less attention to employee behavior and team functioning. This study suggests that digital transformation is more likely to produce innovation outcomes when digital leadership activates task innovation behavior and when the team context supports this behavior. Team collaboration climate provides the communication, knowledge sharing, and support needed for innovation attempts to continue. Organizations should therefore integrate technology upgrading, leadership development, and employee task innovation support, while improving team collaboration mechanisms. This alignment may help organizations obtain greater innovative value from digital investments while supporting employee participation and adaptation during digital transformation.

### Limitations and future research

Several limitations should be considered when interpreting these findings. First, the study focuses on task innovation behavior as one behavioral pathway between digital leadership and employee creativity. This focus helps explain how employees respond to digital leadership through task-level efforts to improve work methods and develop new solutions, but other cognitive, motivational, and social processes may operate alongside this pathway (Anderson et al., 2014). Future research could examine complementary processes involving digital self-efficacy, psychological empowerment, knowledge sharing, or job crafting, as well as strain-related responses such as technostress and cognitive overload. The internal consistency of the task innovation behavior measure was comparatively modest, which may have reduced the precision of associations involving this construct. Replication with further validated task-focused measures would help assess the robustness of the observed pattern.

Second, the contextual conditions examined here are necessarily selective. Team collaboration climate captures an important aspect of the immediate social environment, but the effects associated with digital leadership may also vary with organizational digital maturity, technological infrastructure, task characteristics, and employees’ digital competence (Cortellazzo et al., 2019). The Chinese organizational setting provides a relevant context for examining leadership during ongoing digital transformation, while leaving open whether similar patterns emerge in other institutional and cultural environments. Future research could therefore consider additional team and organizational conditions and assess the model across industries, regions, and cultural contexts.

Third, the sampling strategy and research design place some boundaries on the interpretation of the findings. Participants were recruited primarily through MBA classes and alumni networks at one university in Jinan using convenience sampling. Although they were full-time employees working across different organizations and industries, the sample may not fully reflect the diversity of the broader workforce and included a somewhat higher proportion of women. Future research could examine the model using larger, multi-site samples with greater variation in demographic and occupational characteristics. In addition, the two-stage, multi-source design reduced concerns associated with relying on a single source for all focal variables, but the nonexperimental nature of the study does not establish causal ordering among digital leadership, task innovation behavior, and employee creativity. Longitudinal or experimental research could provide stronger evidence on how these relationships develop over time.

## Conclusion

This study examined the relationship between digital leadership and employee creativity by considering task innovation behavior as a mediating pathway and team collaboration climate as a boundary condition. The findings indicate that task innovation behavior represents an important task-level pathway, and this pathway becomes stronger as team collaboration climate increases. Together, the results show that the digital leadership-creativity link involves both employees’ task-level innovation efforts and the collaborative context in which these efforts occur.

## Data Availability

The raw data supporting the conclusions of this article will be made available by the authors, without undue reservation.
